# A randomized double-blind, placebo-controlled trial of ganaxolone in children and adolescents with fragile X syndrome

**DOI:** 10.1186/s11689-017-9207-8

**Published:** 2017-08-02

**Authors:** Andrew Ligsay, Anke Van Dijck, Danh V. Nguyen, Reymundo Lozano, Yanjun Chen, Erika S. Bickel, David Hessl, Andrea Schneider, Kathleen Angkustsiri, Flora Tassone, Berten Ceulemans, R. Frank Kooy, Randi J. Hagerman

**Affiliations:** 1 Medical Investigation of Neurodevelopmental Disorders (MIND) Institute, University of California, Davis Medical Center, 2825 50th Street, Sacramento, CA 95817 USA; 2 University of California, Davis School of Medicine, Sacramento, CA USA; 3 Department of Medical Genetics, University of Antwerp, Antwerp, Belgium; 4 Department of Neurology-Pediatric Neurology, University Hospital Antwerp, University of Antwerp, Antwerp, Belgium; 5 Department of Medicine, University of California, Irvine School of Medicine, Orange, California USA; 6 Biostatistics Institute for Clinical and Translational Science, University of California, Irvine, California USA; 7 Seaver Autism Center for Research and Treatment, Department of Genetics and Genomic Sciences, Psychiatry, and Pediatrics, Icahn School of Medicine at Mount Sinai, New York, NY USA; 8 Department of Pediatrics, University of California, Davis Medical Center, Sacramento, CA USA; 9 Department of Psychiatry and Behavioral Sciences, University of California, Davis Medical Center, Sacramento, CA USA; 10 Department of Biochemistry and Molecular Medicine, School of Medicine, University of California, Davis, Sacramento, CA USA

**Keywords:** Fragile X syndrome, Ganaxolone, Clinical trial, Children, Adolescents

## Abstract

**Background:**

Gamma-aminobutyric acid (GABA) system deficits are integral to the pathophysiologic development of fragile X syndrome (FXS). Ganaxolone, a GABA_A_ receptor positive allosteric modulator, is hypothesized to improve symptoms such as anxiety, hyperactivity, and attention deficits in children with FXS.

**Methods:**

This study was a randomized, double-blind, placebo-controlled, crossover trial of ganaxolone in children with FXS, aged 6–17 years.

**Results:**

Sixty-one participants were assessed for eligibility, and 59 were randomized to the study. Fifty-five participants completed at least the first arm and were included in the intention-to-treat analysis; 51 participants completed both treatment arms. There were no statistically significant improvements observed on the primary outcome measure (Clinical Global Impression-Improvement), the key secondary outcome measure (Pediatric Anxiety Rating Scale-R), or any other secondary outcome measures in the overall study population. However, post-hoc analyses revealed positive trends in areas of anxiety, attention, and hyperactivity in participants with higher baseline anxiety and low full-scale IQ scores. No serious adverse events (AEs) occurred, although there was a significant increase in the frequency and severity of AEs related to ganaxolone compared to placebo.

**Conclusions:**

While ganaxolone was found to be safe, there were no significant improvements in the outcome measures in the overall study population. However, ganaxolone in subgroups of children with FXS, including those with higher anxiety or lower cognitive abilities, might have beneficial effects.

**Trial registration:**

ClinicalTrials.gov, NCT01725152

## Background

Fragile X syndrome (FXS) is the most common inherited form of intellectual disability, with prevalence rates estimated at 1 in 4000 males and 1 in 8000 females [1]. It is characterized by a wide spectrum of problems including attention deficits, anxiety, hyperactivity, and autism spectrum disorder (ASD). The etiology of FXS is due to a cytosine-guanine-guanine (CGG) repeat expansion (>200 repeats) in the *FMR1* gene leading to methylation and a reduction or absence of its product, fragile X mental retardation protein (FMRP). Females typically have less severe symptoms compared to males [2] due to the second X chromosome potentially contributing to an appreciable amount of FMRP, attenuating the behavioral and cognitive deficits in FXS.

FMRP is heavily expressed in neuronal tissue [3] and affects the stability, localization, and translation of hundreds of mRNAs [4]. Consequently, its absence leads to the dysregulation of several molecular pathways. For example, the metabotropic glutamate receptor 5 pathway (mGluR) is overactive in the absence of FMRP leading to excessive mGluR-mediated long-term depression (LTD) [5]. This knowledge has paved the way for the development of targeted treatments, and preclinical trials using mGluR5 negative allosteric modulators have successfully rescued the FXS phenotype in animal models [6]. Controlled trials in human participants, however, have failed to demonstrate efficacy [7–10].

Another promising avenue for targeted treatments in FXS is the gamma-aminobutyric acid (GABA) system, which provides inhibitory control in the central nervous system (CNS). GABAergic dysfunction has been demonstrated in both animal models and human patients [11–16], and this is believed to contribute to many of the behavioral problem characteristics of FXS [17]. The GABA system acts through two main classes of receptors: GABA_A_ and GABA_B_. GABA_A_ receptors are comprised of multiple subunits assembled into subtypes with unique physiological characteristics and expression patterns throughout the CNS (as reviewed in [17]). Extrasynaptic GABA_A_ receptor subtypes containing a δ-subunit—which provide persistent tonic inhibitory control compared to fast phasic input from other receptor subtypes—are particularly decreased in the neocortex, hippocampus, and cerebellum of the *Fmr1* knock out (KO) mouse. Positron emission tomography (PET) studies have also shown decreased GABA_A_ receptor availability in the human FXS brain [14], with animal studies suggesting these deficits are more pronounced at younger ages [15, 18–20].

Trials of GABA_A_ agonists in FXS animal models have shown positive effects at the cellular and behavioral levels. Treatment with alphaxolone, a neuroactive steroid that targets GABA_A_ receptors, reduced anxiety and rescued audiogenic seizures in the *Fmr1* KO mouse [21]. Gaboxadol—a GABA_A_ receptor superagonist that specifically targets receptors containing the δ-subunit—restored neuronal excitability in the amygdala to normal levels [22] and has mitigated hyperactive behaviors and prepulse inhibition (PPI) deficits [23]. Ganaxolone, a β-methylated synthetic analog of the neuroactive steroid allopregnanolone, acts as a positive allosteric modulator of GABA_A_ receptors [24–26], with its greatest effects on GABA_A_ receptor subtypes containing the δ-subunit. Additionally, because of the β-methylation, ganaxolone is not broken down to additional active metabolites and thus lacks systemic hormonal side effects [24]. Following oral administration, the terminal half-life of ganaxolone is approximately 20 h [27]. It is a known anxiolytic in animal models [28] and has been well tolerated in both adult and pediatric populations in the treatment of epilepsy and traumatic stress disorder [29, 30]. Preclinical trials of ganaxolone in FXS animal models showed a reduction of audiogenic seizures [21] and a dose-dependent reduction in stereotypic and repetitive behavior [15]. These background data led to this investigative study to determine the safety and efficacy of ganaxolone in children with FXS ages 6–17 years old, as well as to measure the effects of ganaxolone on attention, anxiety, hyperactivity, and social behavior in this population.

## Methods

### Experimental design

This was a double-blind, crossover-controlled treatment trial of ganaxolone, a rational translational treatment for children with FXS. Its objective was to assess if ganaxolone was safe to use in children with FXS aged 6–17 years and determine the effect of ganaxolone on behavior. Participant inclusion criteria were a molecular diagnosis of FXS (more than 200 CGG repeats methylated or partially methylated in the *FMR1* gene), age at consent between 6 to 17 years, and willingness to participate in the study. Both male and female participants were included in the study. Sexually active females of child bearing potential were required to have a medically acceptable method of birth control and a negative pregnancy test at initial screening. Exclusion criteria included concomitant use of steroids; active CNS infection or comorbid degenerative neurological disease; aspartate aminotransferase (AST), alanine aminotransferase (ALT), or total bilirubin greater than four times the upper limit of normal (ULN); history of status epilepticus or exposure to any other investigational drug within 30 days prior to randomization.

Participants were recruited through local advertisements and the University of California, Davis (UCD) MIND Institute from November 2012 to June 2015. Potential participants were screened by telephone using a questionnaire for inclusion and exclusion criteria. Those who met all criteria were scheduled for a baseline visit. All families signed an informed consent that was reviewed by the UCD Institutional Review Boards (IRBs). Recruitment at the Belgium site occurred from November 2014 to April 2015 via the Antwerp and Leuven University Hospitals and the Belgian and Dutch fragile X patient organizations. The last participants completed the study in October 2015.

**Fig. 1:**
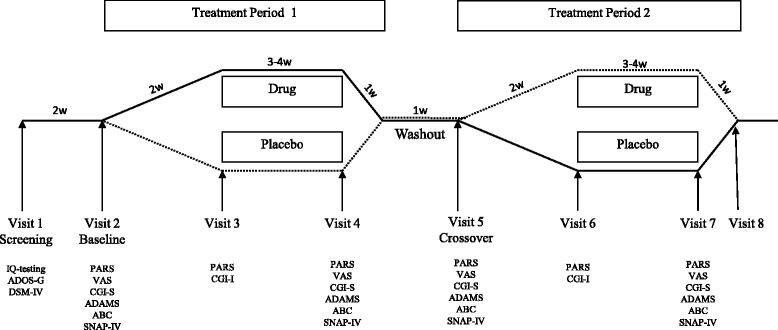
Outline of the study design

The study was designed to have 90% power to detect a modest effect size of 0.6 on the primary outcome measure (CGI-I) at level 0.05. A total of 60 patients were required in a two-treatment crossover design. There are two important aspects to the sample size design: (1) the variation in outcome (CGI-I) measurements and (2) the change or improvement in the outcome between treatment groups that is clinically meaningful. Our design assumptions were that (1) the standard deviation in difference of CGI-I was 1.5 and (2) that we aimed to detect an average 1-point improvement on the CGI-I between treatment groups. These assumptions translate to an effect size of 0.67, and our study was designed to detect an effect size of 0.6. With respect to the CGI-I outcome, a 1-point improvement on average may be going from “no change” to “minimally improved,” for instance. The study design and outcome measures used between UCD and Belgium were similar with the exception of a 1-week difference in duration of stable dose of medication during each arm of the study. In total, participants at the UCD site received 12 weeks of treatment (2-week up-titration and 4-week stable dose per arm) with 1-week down-titration and 1-week washout between treatment arms. Participants at the Belgium site received 10 weeks of treatment (2-week up-titration and 3-week stable dose per arm) with 1-week down-titration and 1-week washout period between treatment arms (Fig. 1). Based on the pharmacokinetics half-life of ganaxolone, a 1-week washout was considered sufficient for ganaxolone to be effectively eliminated from the subject. Subject evaluations took place at baseline and at the end of each treatment period. For some select assessment measures, evaluation also occurred at the mid-period to allow for additional measurements of key study outcomes.

Study drug randomization was completed through the UCD Medical Center Investigational Drug Service and the Pharmacy of the Antwerp University Hospital, independent of the study team. The research teams were blinded until the last participant completed the study. Study drug was prescribed to participants, administered as an oral suspension, in increasing increments of 9 mg/kg/day until a maximum tolerated dose was reached. The maximum allowable dose was 36 mg/kg/day or a maximum of 1800 mg a day. The placebo suspension consisted of the same components except ganaxolone.

Medication compliance was monitored through daily dosing diaries and compared with the amount of remaining drug at each visit. Safety assessments included vital signs, physical exams completed by study physicians, electrocardiogram evaluations, urinalysis, and blood labs including a comprehensive metabolic panel and complete blood count with differential at the beginning and end of each treatment period. Sparse samples of plasma were obtained to determine ganaxolone levels.

Baseline assessments included intelligence testing (Stanford-Binet Intelligence Scales; Wechsler Intelligence Scale for Children (WISC-III); Wechsler Preschool and Primary Scale of Intelligence (WPPSI-III); Wechsler Nonverbal Scale of Ability; Bayley Scales of Infant Development II (BSID-II-NL); or the Snijders-Oomen Non-Verbal Intelligence Test (SON-R; [31]); Autism Diagnostic Observation Schedule (ADOS); Diagnostic and Statistical Manual of Mental Disorders, Text Revision IV (DSM-IV) checklist; and Clinical Global Impression-Severity (CGI-S)).

The primary outcome measure was the Clinical Global Impression-Improvement (CGI-I). The key secondary outcome measure was the Pediatric Anxiety Rating Scale-R (PARS-R; [32]). Other study assessments, administered at baseline and follow-up visits, included the Visual Analogue Scale (VAS) with pre-specified target behaviors of anxiety, attention, hyperactivity, sociability, language, and stereotypic behavior; Anxiety, Depression and Mood Scale (ADAMS; [33]); Aberrant Behavior Checklist-Community Edition (ABC-C), ABC-C FXS algorithm (ABC-C_FXS_) subscales [34]; and Swanson, Nolan and Pelham Questionnaire, 4th edition (SNAP IV; [35]).

### Statistical analysis

Efficacy was assessed via a linear mixed-effect (LME) model for repeated measures in a ganaxolone/placebo, 2-period crossover trial with primary endpoint at the end of the period. The model terms included treatment, time, treatment-by-time interaction, period, treatment-by-period interaction, and baseline measurement if available. Estimation was based on restricted maximum likelihood, and the test denominator degrees of freedom was based on the Kenward-Roger approximation. An unstructured within-subject covariance was used. Potential carryover effect was examined with treatment × period interaction in the model. Analysis of secondary measures follows the same approach with no adjustments for multiplicity. In post hoc analyses, we examined treatment effects with respect to primary and secondary outcomes in five post hoc analyses that restrict to participants (1) with ASD, (2) with very low intellectual functioning (full scale IQ ≤ 45), (3) younger children (ages 6–9), (4) with higher baseline anxiety (≥13 on baseline PARS-R total score), and (5) on concomitant medications minocycline, sertraline, or aripiprazole. All tests were conducted at significance level of 0.05. Due to the large number of secondary measures and post hoc analyses and because these analyses are considered exploratory, we reported results with *p* < 0.1 for all other secondary measures to improve readability of the tables of results. Characteristics of participants in each treatment sequence were compared using *t* test and Fisher’s exact test for continuous and categorical variables, respectively. AEs were summarized by type and severity. We compared prevalence of any moderate/severe AEs, drug-related AEs, and resolution of AEs on a per-person basis using generalized linear mixed model accounting for repeated measurements across period; analyses based on the count of AEs as the outcome resulted in the same conclusion (results not reported). Analyses were implemented in SAS ® software Version 9.4 (SAS Institute Inc.; Cary, NC USA).

## Results

### Demographics and subject disposition

**Fig. 2:**
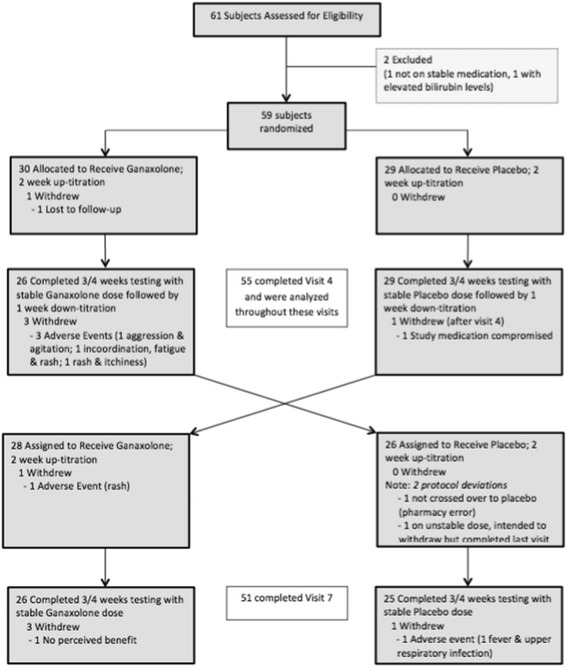
Consort diagram. Disposition of study participants. The intention-to-treat population consisted of all participants who had at least one post-baseline measurement on the primary outcome (CGI-I) and received at least one dose of medication (*n* = 55)

Sixty-one participants were assessed for eligibility, 59 of which were randomized into the two-treatment sequence (Fig. 2). Forty-eight participants were from the UCD site, and 11 from the University Hospital Antwerp, Belgium site. Fifty-five participants (90.2%) completed at least the first period of the study and were included in the modified intention-to-treat (ITT) population analysis of efficacy. This is not strictly an ITT population because 4 subjects randomized and dropped out at the start of the study did not have outcome data for analysis. This included one subject who did not cross over to the placebo treatment after receiving ganaxolone (i.e., received ganaxolone in both periods due to pharmacy error) and a second subject who intended to withdraw but continued on to complete the last visit while on unstable dose. Fifty-one participants (86.4%) completed the second period.

The demographic characteristics of the 59 patients are shown in Table 1. There was no significant difference between the two treatment sequences with respect to demographic characteristics. The majority of participants in both treatment sequences were boys, of white race, not Hispanic or Latino, taking concomitant medications (listed in Table 1; 93% in both groups), and an average age of 11.3 years (placebo-ganaxolone) and 10.6 years (ganaxolone-placebo) at baseline visit.

**Table 1:** Demographic and clinical characteristics of 59 participants, randomly assigned to placebo-ganaxolone and ganaxolone-placebo

	Placebo-ganaxolone			Ganaxolone-placebo			
Variable	*N*	Mean	SE	*N*	Mean	SE	*p* value
Age at visit 1	29	11.3	0.6	30	10.6	0.6	0.40
FSIQ standard score	23	54.3	4.5	26	48.4	2.3	0.24
	*N*	%		*N*	%		
Gender							
Male	23	79.3		27	90.0		0.25
Female	6	20.7		3	10.0		
Race							
Asian	2	6.9		2	6.7		0.23
Black or African American	0	0.0		4	13.3		
White	24	82.8		22	73.3		
More than one race	3	10.3		2	6.7		
Ethnicity							
Hispanic or Latino	5	17.2		5	16.7		0.58
Not Hispanic or Latino	23	79.3		25	83.3		
Unknown/not reported	1	3.4		0	0.0		
Concomitant medication							
No	2	6.9		2	6.9		1.00
Yes	27	93.1		27	93.1		
Concomitant medication details^a^							
Aripiprazole	4	13.8		5	17.2		0.72
Antioxidants	2	6.9		1	3.4		0.55
Minocycline (Dynacin/Minocin)	10	34.5		8	27.6		0.57
Ritalin/Concerta/Methylin/Metadate/Methylphenidate	3	10.3		4	13.8		0.69
Valproic acid (Depakote/Depakene/Epilim/Stavzor)	2	6.9		3	10.3		0.64
Sertraline (Zoloft)	7	24.1		8	27.6		0.76
Other medication	26	89.7		23	79.3		0.28

Demographics of intention-to-treat population. There were no statistically significant differences between patients randomized to placebo-ganaxolone and ganaxolone-placebo treatment sequences

^a^Concomitant medication is based on 58 patients, 1 patient is completely missing concomitant medication information

*SE* standard error

### Primary outcome measure

**Fig. 3:**
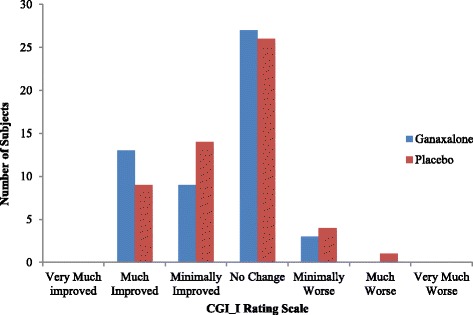
Distribution of Clinical Global Impression-Improvement (CGI-I) Score at end of treatment. Comparison of CGI-I results between ganaxolone and placebo treatment arms in intention-to-treat population (*n* = 55). There was no statistically significant difference in CGI-I scores between treatment arms (*p* = 0.45)

There was no statistically significant difference in the CGI-I for ganaxolone treatment (3.4 ± 0.13) compared to placebo (3.5 ± 0.13). See details in Table 2 and Fig. 3, where the distribution of CGI-I score at end of the treatment is presented. The treatment × period interaction was not significant; thus, the data do not support a possible carryover effect. Because the majority of participants (44 of 55) for the primary efficacy analysis were from UCD, we also examined robustness of the reported results based on the data from the UCD site alone. The overall results and conclusions remained the same.

**Table 2:** Primary and secondary outcome analysis

	Ganaxolone					Placebo					
	Baseline			End of treatment		Baseline			End of treatment		
	*N*	Mean	SE	Lsmean	SE	*N*	Mean	SE	Lsmean	SE	*p* value
Primary outcome											
Clinical Global Impression-Improvement	52	–	–	3.4	0.13	54	–	–	3.5	0.13	0.45
Key secondary outcome											
Pediatric Anxiety Rating Scale (total score)	55	10.5	0.76	8.3	0.54	55	10.9	0.73	9.2	0.54	0.22
Other secondary outcome											
Visual Analogue Scale											
Severity of Anxiety (cm)	55	4.3	0.37	5.6	0.27	54	4.2	0.34	5.0	0.26	0.13
Severity of attention (cm)	55	3.4	0.30	4.4	0.24	54	3.3	0.31	3.9	0.24	0.14
Severity of one selected additional target behavior (sociability, language, aggression, hyperactivity/impulsivity) (cm)	55	3.2	0.30	4.4	0.28	54	3.3	0.33	3.9	0.27	0.25
Anxiety, Depression and Mood Scale											
Manic/hyperactive behavior total	54	8.1	0.51	7.4	0.42	53	8.7	0.50	7.8	0.40	0.49
Depressed mood total	55	3.0	0.46	3.5	0.32	54	3.1	0.42	2.6	0.32	0.01
Social avoidance total	55	6.9	0.60	5.8	0.42	54	7.5	0.62	6.3	0.41	0.39
General anxiety total	55	7.9	0.62	6.2	0.45	54	8.3	0.60	7.0	0.44	0.21
Obsessive/compulsive behavior total	55	3.0	0.32	2.7	0.21	54	3.4	0.31	2.8	0.20	0.81
Aberrant Behavior Checklist-Community Edition FXS algorithm (ABC-C_FX_)											
Total - subscale I (irritability)	55	18.9	1.53	15.7	0.87	54	19.6	1.51	16.1	0.86	0.62
Total - subscale II (lethargy)	55	6.6	0.68	5.6	0.53	52	6.9	0.68	5.7	0.54	0.74
Total - subscale III (stereotypy)	55	7.4	0.68	5.7	0.40	54	7.1	0.68	6.4	0.39	0.17
Total - subscale IV (hyperactivity)	54	13.9	1.00	11.3	0.65	54	13.9	1.14	12.3	0.62	0.28
Total - subscale V (inappropriate speech)	55	6.0	0.47	5.1	0.32	54	6.3	0.43	5.3	0.31	0.47
Total - subscale VI (social avoidance)	55	3.5	0.43	2.4	0.22	54	3.1	0.40	2.8	0.21	0.25
Swanson, Nolan, and Pelham-IV Questionnaire											
ADHD inattention total	53	15.7	0.76	15.5	0.66	53	16.7	0.79	14.6	0.62	0.28
ADHD inattention average	53	1.7	0.08	1.7	0.07	53	1.9	0.09	1.6	0.07	0.28
ADHD hyperactive impulsive total	54	13.0	0.93	13.9	0.66	53	14.0	0.92	12.6	0.64	0.12
ADHD hyperactive impulsive average	54	1.4	0.10	1.5	0.07	53	1.6	0.10	1.4	0.07	0.14
ADHD combined total	53	28.7	1.52	29.3	1.19	53	30.7	1.54	27.1	1.14	0.15
ADHD combined average	53	1.6	0.08	1.6	0.07	53	1.7	0.09	1.5	0.06	0.21

Analysis of study measures in overall participant population (*N* = 55 patients who completed at least one period). There were no statistically significant differences in primary or key secondary measures. However, there was statistically significant increase (worsening) on the ADAMS depressed mood subscale

*ADHD* attention-deficit hyperactivity disorder, *Lsmean* least squares mean; *SE* standard error

### Secondary outcome measures

The key secondary outcome measure was the PARS-R, which measured the number, frequency, and severity of anxiety symptoms. No statistical difference was found between ganaxolone and placebo for the PARS-R total score.

The other secondary outcome measures included the VAS, ADAMS, ABC-C, ABC-C_FX_ subscales [34], and SNAP-IV. For all tests, a lower score indicates an improvement, except for the VAS where a higher score corresponds with a reduced severity of the target behavior. For the ADAMS, the depressed mood subscore was higher at the end of ganaxolone treatment than placebo periods (3.5 ± 0.32 vs. 2.6 ± 0.32, *p* = 0.01). Itemized analysis for the ADAMS depressed mood subscore revealed that the difference was mainly attributed to increased sleep (ganaxolone 0.6 ± 0.10 vs. placebo 0.2 ± 0.10, *p* = 0.01) and less energy (ganaxolone 0.6 ± 0.08 vs. placebo 0.4 ± 0.08, *p* = 0.03). No other measurement showed statistically significant difference between ganaxolone treatment and placebo periods. Details are given in Table 2.

### Safety and tolerability of ganaxolone

There were 334 adverse events (AEs) reported. The top three types of AEs were upper respiratory infection, fatigue, and drowsiness. Details of the types of AEs are provided in Table 3. Characteristics of AEs by severity, relation to drug, AE status (whether ongoing), and serious AEs are also presented in Table 3. No serious AEs were reported. The vast majority of AEs was mild and with status “recovered.” Among participants reporting AEs, participants receiving ganaxolone reported more moderate/severe AEs than placebo (60 vs. 33%, *p* = 0.01). Also, more drug-related AEs were reported in participants during ganaxolone period than placebo (83 vs. 57%, *p* = 0.01). No significant difference in the resolution status of AEs was found between participants during ganaxolone and placebo periods.

**Table 3:** Characteristics of adverse events during ganaxolone and placebo periods

	Ganaxolone		Placebo	
	*N*	%	*N*	%
Severity				
Mild	124	65.6	116	80.0
Moderate	60	31.8	28	19.3
Severe	5	2.7	1	0.7
Resolution status				
Not recovered	19	10.1	14	9.7
Ongoing	5	2.7	5	3.5
Recovered	163	86.2	125	86.2
Unknown	2	1.1	1	0.7
Frequency				
Continuous	46	27.5	32	24.1
Intermittent	106	63.5	84	63.2
Single	15	9.0	17	12.8
Action taken				
Concomitant medication/therapy	13	6.9	17	11.7
Dose decrease	13	6.9	4	2.8
None	153	81.0	118	81.4
Permanently discontinued	10	5.3	4	2.8
Temporarily discontinued	0	0.0	2	1.4
Relation				
Definitely	2	1.1	0	0.0
Probably	44	23.3	9	6.2
Possibly	62	32.8	52	35.9
Unlikely	33	17.5	32	22.1
Not related	48	25.4	52	35.9
Serious				
No	189	100.0	145	100.0
AE details				
Upper respiratory infection	19	10.1	24	16.6
Fatigue	28	14.8	16	11.0
Drowsiness	23	12.2	6	4.1
Diarrhea	10	5.3	10	6.9
Agitation	4	2.1	7	4.8
Vomiting	7	3.7	7	4.8
Rash	9	4.8	6	4.1
Decreased appetite	8	4.2	2	1.4
Gastrointestinal issues	6	3.2	5	3.4
Headache	5	2.6	5	3.4
Ear infection	2	1.1	4	2.8
Fever	1	0.5	4	2.8
Sleep disturbance	3	1.6	4	2.8
Abnormal vocalizations	5	2.6	3	2.1
Aggression	4	2.1	3	2.1
Skin infection	4	2.1	1	0.7
Hyperactivity	3	1.6	3	2.1
Irritability	2	1.1	3	2.1
Rhinorrhea			3	2.1
Anxiety	3	1.6		
Dizziness	3	1.6	1	0.7
Hypersomnia	3	1.6		
Incoordination	3	1.6	1	0.7
Itchiness	3	1.6		
Disruptive behavior	1	0.5	2	1.4
Self-injurious behavior	2	1.1	2	1.4
Falling	2	1.1	1	0.7
Incontinence	2	1.1	1	0.7
Skin abrasion	2	1.1	1	0.7
Tics	2	1.1		
Abdominal pain upper	1	0.5	1	0.7
Dental trauma			1	0.7
Drooling	1	0.5	1	0.7
Elevated transaminase	1	0.5	1	0.7
Emotional lability			1	0.7
Enlarged aorta			1	0.7
Flushing			1	0.7
Gynecomastia			1	0.7
Hyperphagia			1	0.7
Hypoglycemia			1	0.7
Ketonuria			1	0.7
Menstrual cramps			1	0.7
Nausea	1	0.5	1	0.7
Obsessive Compulsive Behavior			1	0.7
Ruptured Ear Drum			1	0.7
Scratch			1	0.7
Seizures	1	0.5	1	0.7
Staring Spells	1	0.5	1	0.7
Thirst			1	0.7
Wound			1	0.7
Acne	1	0.5		
Decreased fluid intake	1	0.5		
Desquamation	1	0.5		
Dry mouth	1	0.5		
Excess cerumen	1	0.5		
Eye infection	1	0.5		
Increased appetite	1	0.5		
Musculoskeletal injury	1	0.5		
Nervousness	1	0.5		
Pallor	1	0.5		
Pharyngitis	1	0.5		
Rhinorrhea	1	0.5		
Thermal burn	1	0.5		
Dental operation	1	0.5		

Analysis of adverse events (AEs) in intention-to-treat population. No significant AEs occurred throughout the study, although there was a higher number and severity of AEs while participants were taking ganaxolone compared to placebo. There was a higher incidence of fatigue and drowsiness, and this is believed to be due to a sedative effect of ganaxolone

### Post hoc analyses

#### Participants with higher baseline anxiety

**Fig. 4:**
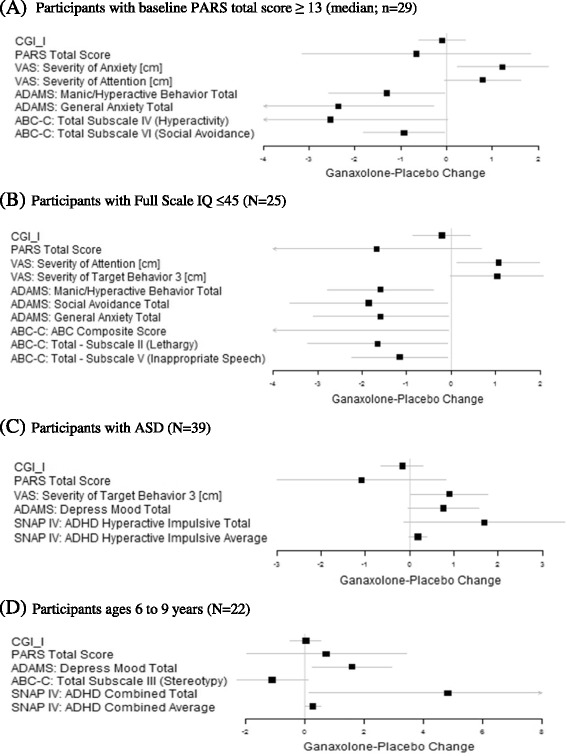
Post hoc analyses of primary and secondary measures. Post hoc analysis in subpopulations of children with FXS. Difference at end of treatment periods, adjusted for baseline (*square*), and 95% confidence interval (*line*). Positive scores on the VAS indicate improvement. For all other measures, negative scores indicate improvement. No subgroups showed statistically significant effects on primary or key secondary measures. Participants with higher baseline anxiety (**a**) showed positive effects across multiple test measures in areas of anxiety (VAS and ADAMS) and hyperactivity (ADAMS and ABC-C_FX_). Participants with low FSIQ (**b**) showed positive effects across the most behavior areas. Participants with ASD (**c**) and young participants (**d**) showed the modest effects on secondary measures. Only measures with *p* value <0.1 displayed for other secondary outcomes. *ADHD* attention-deficit hyperactivity disorder, *VAS* Visual Analogue Scale, *SNAP IV* Swanson, Nolan, and Pelham-IV Questionnaire, *PARS* Pediatric Anxiety Rating Scale, *ADAMS* Anxiety, Depression and Mood Scale, *CGI-I* Clinical Global Impression-Improvement, *ABC-C* Aberrant Behavior Checklist-Community Edition FXS algorithm (ABC-C_FX_)

Because anxiety is a central hallmark of FXS, we were interested on whether participants with an overall lower level of anxiety would show little improvement in anxiety scores and thus obscure the potential positive effects in higher anxious participants. We conducted a post hoc analysis for participants with higher level of anxiety at baseline, defined as baseline PARS-R greater than or equal to 13 (median score of study population). For patients with PARS-R ≥ 13 (*n* = 29) at baseline, we found no statistically significant difference with respect to the primary and key secondary outcomes between ganaxolone and placebo. For other secondary measures, significance in higher average VAS scores, indicating improvement, were observed for ganaxolone compared to placebo periods with respect to anxiety (5.4 ± 0.32 vs. 4.2 ± 0.31, *p* = 0.02) as well as a positive trend in attention (4.7 ± 0.32 vs. 4.0 ± 0.30, *p* = 0.08). Also, lower scores, demonstrating improvement, were reported for participants receiving ganaxolone compared to placebo in manic/hyperactive behavior (7.4 ± 0.54 vs. 8.7 ± 0.49, *p* = 0.06) and general anxiety improvements (7.3 ± 0.67 vs. 9.7 ± 0.67, *p* = 0.04) in ADAMS, and in hyperactivity (10.8 ± 0.86 vs. 13.3 ± 0.79, *p* = 0.06), and social avoidance (3.2 ± 0.30 vs. 4.1 ± 0.30, *p* = 0.05) in ABC-C_FX_. Differences in outcomes at the end of the treatment periods (ganaxolone-placebo), adjusted for baseline measurement along with 95% confidence intervals are given in Fig. 4a; 95% CIs are displayed as horizontal lines, no difference indicated by the vertical line at zero, and difference in outcomes between treatment periods indicated by the square marks.

#### Participants with full-scale IQ ≤ 45

There were *n* = 25 participants with FSIQ less than or equal to 45. Similarly, no significant difference was found in the primary and key secondary outcomes between ganaxolone and placebo periods. For other secondary measurements, participants with IQ ≤ 45 on ganaxolone had a significant improvement in higher average VAS scores in of attention (4.4 ± 0.35 vs. 3.3 ± 0.34, *p* = 0.04) and a trend for improvement in participant selected target behavior (4.0 ± 0.39 vs. 2.9 ± 0.37, *p* = 0.07). Lower scores, also demonstrating improvement, were reported at the end of the ganaxolone period compared to placebo with respect to manic/hyperactive behavior (8.0 ± 0.59 vs. 9.6 ± 0.58, *p* = 0.02), social avoidance (5.7 ± 0.66 vs. 7.6 ± 0.63, *p* = 0.05), general anxiety (6.5 ± 0.63 vs. 8.0 ± 0.64, *p* = 0.05) on the ADAMS, as well as the ABC-C_FX_ social withdrawal/lethargy subscale (5.7 ± 0.81 vs. 7.4 ± 0.84, *p* = 0.06) and inappropriate speech subscale (5.3 ± 0.43 vs. 6.4 ± 0.42, *p* = 0.05). See Fig. 4b for estimate of treatment differences and 95% CIs.

#### Participants with ASD

For participants with ASD (*n* = 39), no significant difference was found between ganaxolone and placebo periods with respect to the primary and key secondary outcomes. For other secondary measures, participants with ASD receiving ganaxolone had a potential trend of reporting higher average score than participants receiving placebo in improvement in target behavior in VAS (4.3 ± 0.35 vs. 3.4 ± 0.35, *p* = 0.05), depressed mood subscore on the ADAMS (3.4 ± 0.38 vs. 2.6 ± 0.40, *p* = 0.07), and attention deficit hyperactivity disorder (ADHD) hyperactive impulsive total in SNAP-IV (14.7 ± 0.82 vs. 13.0 ± 0.80, *p* = 0.08). See Fig. 4c for estimate of treatment differences and 95% CIs.

#### Younger participants ages 6–9

Similar to the post hoc analysis for participants with ASD and low IQ, for younger participants ages 6–9 years (*n* = 22), no significant difference was found between ganaxolone and placebo period with respect to the primary and key secondary outcomes. Participants on ganaxolone reported higher average depressed mood score (3.6 ± 0.62 vs. 2.0 ± 0.58, *p* = 0.03) on the ADAMS and ADHD combined total score (32.1 ± 1.96 vs. 27.2 ± 1.91, *p* = 0.06) on the SNAP-IV, compared to participants receiving placebo. A lower score, demonstrating improvement, was observed for participants with ganaxolone compared to placebo in stereotypic behavior (5.8 ± 0.53 vs. 6.9 ± 0.52, *p* = 0.09) in ABC-C_FX_. Estimate of treatment differences and 95% CIs are given in Fig. 4d.

#### Participants on concomitant medication minocycline, sertraline, or aripiprazole

Twenty-four participants were on concomitant medications including minocycline, sertraline, or aripiprazole. Similar to the main analyses (see Table 2) of primary efficacy (CGI-I) and key secondary (PARS-R), there were no significant difference between ganaxolone and placebo periods. There was no difference in all other secondary measure as well (results not shown).

### Other sensitivity analyses

Because two participants had major protocol deviations (see Fig. 2), we considered a per-protocol-population analysis that excluded these two participants. The results and conclusion remained the same. Because the majority of the participants were from UCD, we also conducted sensitivity analyses based on the UCD data only. The pattern of results remained the same, with the reported differences in secondary measures above becoming slightly attenuated due to the smaller sample size.

## Discussion

This was a phase 2 double-blind, crossover trial of ganaxolone as a targeted treatment for children with FXS. Ganaxolone is a GABA_A_ positive allosteric modulator that has provided promising results in the FXS mouse model [13, 21] and has a proven safety profile in both children and adults patients in other disease models [29, 30]. This study did not identify any statistically significant improvements in the primary, the key secondary or other secondary outcome measures for children with FXS taking ganaxolone compared to placebo. However, post hoc analysis revealed positive effects in favor of the study medication in subpopulations.

Positive effects in areas of attention, hyperactivity, and anxiety were observed in participants with higher baseline anxiety (PARS-R ≥ 13) and those with low FSIQ scores (IQ ≤ 45) while taking ganaxolone compared to placebo. The higher anxiety group showed reduction in anxiety (VAS and ADAMS) and hyperactivity (ADAMS and ABC-C_FX_) across multiple test measures, suggesting ganaxolone may be helpful for these symptoms. However, there was not a statistically significant improvement on the PARS-R; therefore, the PARS-R was useful as a screening tool to select the higher anxiety subpopulation but appeared to be less sensitive than other outcome measures to monitor ganaxolone treatment response. The low FSIQ subgroup was chosen because longitudinal studies have demonstrated a relationship between cognitive deficits and behavioral problems in FXS [36]; therefore, we later hypothesized participants with the lowest IQ could also have the greatest behavioral deficits, and thus, ganaxolone could be effective in this group. This subpopulation experienced the most positive trends out of all post hoc subgroups in this study, and future research should also consider assessing if behavioral changes correlate with cognitive benefits as well. Overall, the trends observed in these subgroups are encouraging, and further trials of ganaxolone in patients with higher baseline anxiety and those with low FSIQ scores are warranted. There was no obvious correlation of effects with plasma levels of ganaxolone, which were generally similar to those if adults given a dose of 1800 mg/day.

The ASD subgroup was chosen because there is known GABA_A_ergic dysfunction in idiopathic ASD [37, 38], and preclinical trials have demonstrated positive effects of ganaxolone on social behavior in ASD mouse models [39]. Participants with ASD in this study experienced a positive trend in targeted behaviors on the VAS. However, while sociability was one of the target behavior outcomes, there was insufficient data to analyze changes in this area. Related indicators such as the social avoidance subscales on the ADAMS and ABC-C_FX_ subscales were not significantly different between ganaxolone and placebo arms showing that sociability may not be improved by ganaxolone in this subgroup. It would be interesting to study treatment outcomes of ganaxolone in idiopathic autism as there is emerging evidence suggesting differences between nonsyndromic ASD and ASD in FXS [40–43].

Data from the youngest participants (ages 6–9 years) was analyzed because deficits in GABA expression are more pronounced at younger ages [16]. In this group, there was a positive trend indicating decreased stereotypic behaviors while taking ganaxolone. This is congruent with decreased stereotypic behaviors (repetitive and perseverative marble burying) in the FXS mouse models when treated with ganaxolone [15].

The overall patient population experienced statistically significant worsening on the ADAMS depressed mood scale while on ganaxolone compared to placebo. Itemized analysis of the ADAMS revealed this change was due to decreases in energy level and increases in sleep, which are likely associated with the two most common reported AEs of fatigue (14.8% ganaxolone vs. 11% placebo) and drowsiness (12.2% ganaxolone vs 4.1% placebo). This was expected due to the GABAergic mechanism of action. Negative trends were also seen in the depressed mood scale (ADAMS) and ADHD symptoms (SNAP-IV) in the ASD and young age subgroups as well. The worsening of ADHD symptoms in select subgroups may indicate a partial activating effect, which has been observed in previous trials of ganaxolone in other patient populations [27, 29, 30].

Ganaxolone was generally safe, as there were no serious AEs reported during this study. However, there was a statistically significant increase in the number and severity of AEs related to study medication. Future studies should closely monitor these changes, but an overwhelming majority of participants recovered from the reported AEs by the end of the study. Because of the short duration of the study, potential long-term AEs of ganaxolone were not examined, although it is an important issue to consider in future studies.

One of the key limitations of this study was that the positive trends were found during post hoc analyses only. Of these, anxiety and hyperactivity were improved across multiple measures in the higher anxiety group. It is possible that certain outcome measures capture improvements in these areas better compared to others. Second, the study was designed to meet 90% power, but enrollment goals were not met due to resource limitations and the inadequate recruitment at a single site. Recruitment of a second site (Belgium) with a similar protocol helped increase participant numbers, but the two sites varied in number of weeks of stable dose (4 weeks at UCD, 3 weeks at Belgium). This limitation underscores the critical need for multi-site studies particularly for rare or difficult to reach populations. Analysis of results from the UCD only did not show significant differences from the combined data, and it is likely the 1 week difference in protocol had minimal effects on the overall results. We note that although the choice of the crossover design is more efficient than a standard parallel design, a major potential drawback is the loss of subjects. Dropouts were relatively low in this study.

## Conclusions

This study provides information regarding which subgroups of patients should be included in future trials of ganaxolone in FXS. Our data suggests participants with higher anxiety and low FSIQ should be targeted as part of the inclusion criteria. It may also be possible to study ganaxolone in younger patients with FXS since it has been found to be safe at younger ages. Patients with seizures may have additional benefits, as ganaxolone has also shown improvements in children with epilepsy [29]. Additional trials of ganaxolone in children with FXS are warranted, and this study could provide a framework for more targeted inclusion criteria and outcome measures in future trials.

## Abbreviations

ABC-C: Aberrant Behavior Checklist–Community Edition
ADAMS: Anxiety, Depression and Mood Scale
ADHD: Attention deficit hyperactivity disorder
ADOS: Autism Diagnostic Observation Schedule
AE: Adverse event
ALT: Alanine aminotransferase
ASD: Autism spectrum disorder
AST: Aspartate aminotransferase
BSID-II-NL: Bayley Scales of Infant Development II
CGG: Cytosine-guanine-guanine
CGI-I: Clinical Global Impression, Improvement
CGI-S: Clinical Global Impression–Severity
CNS: Central nervous system
DSM-IV: Diagnostic and Statistical Manual of Mental Disorders, Text Revision IV
FXS: Fragile X syndrome
GABA: Gamma-aminobutyric acid
IRB: Institutional review board
ITT: Intention-to-treat
LME: Linear mixed-effect
LTD: Long-term depression
mGluR: Metabotropic glutamate receptor
PARS: Pediatric Anxiety Rating Scale
PET: Positron emission tomography
PPI: Prepulse inhibition
SNAP IV: Swanson, Nolan and Pelham Questionnaire, 4th edition
UCD: University of California, Davis
ULN: Upper limit of normal
VAS: Visual Analogue Scale
WISC-III: Wechsler Intelligence Scale for Children
β-HCG: β-human chorionic growth hormone
